# Editorial: II Bio.natural-bioactive natural products research meeting: Pharmacology perspectives

**DOI:** 10.3389/fphar.2022.991644

**Published:** 2022-09-13

**Authors:** Patricia Rijo, Thomas Efferth

**Affiliations:** ^1^ CBIOS – Universidade Lusófona´s Research Center for Biosciences & Health Technologies, Lisboa, Portugal; ^2^ Research Institute for Medicines (iMed.ULisboa), Faculty of Pharmacy, Universidade de Lisboa, Lisboa, Portugal; ^3^ Department of Pharmaceutical Biology, Institute of Pharmaceutical and Biomedical Sciences, Johannes Gutenberg University, Mainz, Germany

**Keywords:** natural products, pharmacology congress, bioactivity, natural products chemistry, bio.natural meeting

After the success of the first international Meeting Bio. Natural (Bioactive Natural Products Research Meeting - 2019), held on 2019 at Lisbon, Portugal, the organization lauched the second edition of the event in 2021. *The II Bio. Natural Meeting–Bioactive Natural Products Research Meeting* was helded virtually on 18th and 19th of November 2021 (Universidade Lusófona de Humanidades e Tecnologias, Lisbon, Portugal). Once again, the meeting aimed to be a forum for researchers who are developing projects exploring the multiple applications offered by natural products. Natural products and their biological and pharmacological effects based on people’s use is important as a source of therapeutic agents. The medicinal substances such as extracts, or isolated compounds are thus valuable tools for drug development or elucidation of the structural requirements for potential clinical application.

In this Research Topic of Frontiers in Pharmacology, we would like to emphasize the high value of natural products, focus only on pharmacological aspects: natural products in drug discovery; natural products chemistry; bioactivity of natural products; marine natural products; functional foods and food supplements, and other fields related to natural products.

This Research Topic collects six contributions, two reviews and four original articles, highlighting the potential use of natural products in pharmacology studies on papers selected from “ *II Bioactive Natural Products Research Meeting - II Bio. Natural Meeting 2021*”.

An original research work by Wang et al. focused on the *in vivo* detoxification method and mechanism of Semen Strychni acute poisoning and the alleviation effects of isoliquiritigenin. The results indicated that Semen Strychni induced neuronal degeneration in the hippocampal CA1 region, and inhibited the mRNA expression of NMDAR1, NMDAR2A, NMDAR2B, GABRa1, GABRb2 and reduced the level of MAO, which disrupted the DA and 5-HT metabolic pathway. However, isoliquiritigenin reversed these effects. In summary, isoliquiritigenin showed alleviation effects on Semen Strychni-induced neurotoxicity, which could be attributed to restoring neurotransmitters metabolic pathway, most likely through the activation of NMDA receptors.

The original research work by Pyun et al. summarized reports of how dTCTP’s role in allergic inflammation can be modulated or negated, and the possible potential of cardamonin as an anti-allergic agent. The interaction between cardamonin and dTCTP was confirmed by SPR assay. Cardamonin was found to reduce the secretion of IL-8 caused by dTCTP in BEAS-2B cells by interacting with dTCTP. Cardamonin reduced the migration of various inflammatory cells in the bronchoalveolar lavage fluid (BALF), inhibited OVA specific IgE secretion and bronchial remodeling. In addition, cardamonin was observed to have an anti-allergic response by inhibiting the activity of NF-κB. Cardamonin exerts anti-allergic anti-inflammatory effect by inhibiting dTCTP, suggesting that it may be useful in the therapy of allergic diseases.

The review work by Singla et al. summarizes the genus *Alternanthera*: Phytochemical and Ethnopharmacological Perspectives that is used traditionally for the treatment of various ailments such as hypertension, pain, inflammation, diabetes, cancer, microbial and mental disorders. In conclusion, the available literature searched on pharmacological studies of *Alternanthera* species reveals that few species have been selected based on ethnobotanical surveys for scientific validation of their traditional claims. But most of these studies have been conducted on uncharacterized and non-standardized crude extracts. A roadmap of research needs to be developed for the isolation of new bioactive compounds from *Alternanthera* species, which can emerge out as clinically potential medicines.

In turn, the original work by Cheng et al. focused on Natural Compound Library Screening Identifying Oroxin A as the Treatment of Myocardial Ischemia/Reperfusion Injury. The aim of this study was to determine whether OA could alleviate MI/RI induced inflammation and pyroptosis *in vivo* and *in vitro*, providing a novel therapeutic regimen for the treatment of MI/RI. The results revealed that OA is an effective remedy against MI/RI as it reduces the inflammatory response and inhibits pyroptosis. This may provide a new therapeutic target for the clinical treatment of MI/RI.

The original research work by Ma et al. reported the A Novel Small-Molecule Inhibitor of SREBP-1 Based on Natural Product Monomers that Upregulates the Sensitivity of Lung Squamous Cell Carcinoma Cells to Antitumor Drugs. The novel small-molecule inhibitor of SREBP-1, MSI-1 (Ma’s inhibitor of SREBP-1), based on natural product monomers, was identified by screening the database of natural products. Treatment with MSI-1 suppressed the activation of SREBP-1-related pathways and the Warburg effect of LUSC cells, as indicated by decreased glucose uptake or glycolysis. Moreover, the treatment of MSI-1 enhanced the sensitivity of LUSC cells to antitumor agents. The specificity of MSI-1 on SREBP-1 was confirmed by molecular docking and point-mutation of SPEBP-1. Therefore, MSI-1 improved our understanding of SREBP-1 and provided additional options for the treatment of LUSC.

The review work by Zhou et al. studied the therapeutic Effects of Natural Products on Cervical *Cancer*, Based on Inflammatory Pathways. Natural products are considered excellent candidates for the treatment of cervical cancer. In this review, it was first described the mechanisms by which inflammation induces cervical cancer. In addition, it was highlight that natural products can treat cervical cancer through inflammatory pathways and also introduced natural products for the treatment of cervical cancer in clinical trials. Finally, methods to improve the anticancer properties of natural products were added, and the development status of natural products was discussed.

Overall, the works presented in this Research Topic provide experimental evidence and assembled scientific data, which clearly emphasize the potential use of natural products in pharmacology studies on papers selected from “ *II Bioactive Natural Products Research Meeting - II Bio. Natural Meeting 2021*”.

